# Supplementary material for: Are carbon emissions trading and green financial instruments synergistic? -Comprehensive quantitative research based on content analysis
Source: PLoS One. 2024 Mar 7;19(3):e0298601. doi: 10.1371/journal.pone.0298601 (PMC10919601; doi:10.1371/journal.pone.0298601)
Supplement: S1 Data — (ZIP) [file pone.0298601.s001.zip › Data/地级市Z_F工作报告.zip!/║■▒▒/╗╞╕╘╩╨/└┤╫╘╣1⁄2╓┌║┼í╛┬φ┐╦╩2╛▌═°í┐_2008.docx]

各位代表：

　　现在，我代表市人民政府，向大会作政府工作报告，请予审议，并请市政协各位委员和其他列席人员提出意见。

　　一、2007年工作回顾

　　2007年，在市委正确领导下，市人民政府深入贯彻落实科学发展观，团结带领全市人民，抢抓机遇，扎实工作，市三届人大一次会议确定的主要预期目标全面超额完成，经济建设、政治建设、文化建设和社会建设取得显著成就。

　　经济发展跃上新台阶。全市地区生产总值473.7亿元，比上年增长14.4%。财政收入45.3亿元，比上年增长31.3%。其中地方一般预算收入19.9亿元，比上年增长29.8%。城镇以上固定资产投资、社会消费品零售总额、外贸出口等3项指标增幅和规模以上工业企业新增数量居全省首位。农业和农村经济出现可喜变化。主要农产品全面增产，棉花产量、生猪出栏、家禽出笼、肉牛出栏、肉羊出栏、水产品产量创历史新高。奶牛养殖、“虾稻连作”、“林水结合”和市区“菜篮子”工程建设成为农村经济发展的新亮点。创建中国地理标志保护产品2个、全国劳务品牌3个、国家级农业标准化示范区6个。工业经济增势强劲。规模以上工业企业发展到785家，当年新增186家;工业增加值84.9亿元，比上年增长30.1%。创建国家特级建筑资质企业1个、中国名牌产品2个、国家免检产品5个、湖北名牌产品10个。现代服务业发展提速。完成社会消费品零售总额223.7亿元，比上年增长21.3%。首届大别山农产品进超市对接活动圆满成功。“万村千乡市场工程”覆盖率达51.7%。居民消费价格涨幅为4.46%。金融机构各项存款余额突破500亿元，各项贷款净增37.4亿元。“驻鄂银行行长黄冈老区行”取得实效。房地产业加快发展，全市完成房地产投资23.3亿元，比上年增长91.7%。黄冈大别山旅游品牌促销初见成效，旅游接待人数超过500万人次。烟草、石油、保险、通信等行业发展规模和经营绩效名列全省前茅。

　　城乡面貌发生新变化。全社会固定资产投资258.5亿元，比上年增长34%。交通建设，完成通村油路3316公里，实现100%的乡镇、71%的行政村通沥青(水泥)路。沪汉蓉快速铁路、武英、大广北高速公路建设快速推进。能源建设，大别山电厂一期工程即将并网发电，白莲河抽水蓄能电站按计划有序施工。500KV黄冈输变电工程建成投入运营。农田水利建设，黄广大堤加固及配套建设工程通过省级验收，19座大中型水库整险加固工程完成投资2.7 亿元，高产农田建设完成投资1.3 亿元，总投资4.2亿元的长江回水堤防工程全面启动。新建沼气池3万口。解决了36.8万人饮水困难。信息建设，实施了广播电视、电话“村村通工程”和“农村信息化示范工程”，实现100%的乡镇和80%的行政村宽带互联网接入。城乡建设，启动了市区创建省级园林城市、省级卫生城市、省级文明城市和中国优秀旅游城市“四城联创”工作，投入2.1亿元，实施了“一湖九路”建设工程。加强12个省级示范镇、96个省级试点村、124个市级示范村、200个县级示范村的新农村建设，新建和改造村级办公场所752个。

　　改革开放实现新突破。农村综合改革、集体林权制度改革、国有农场管理体制改革以及粮食流通、农村卫生等体制改革全面展开。全市落实各项惠农补贴资金24011万元，筹措农村公益性服务“以钱养事”资金8246万元。农村专业合作组织发展到323个。部门预算制度渐趋规范，税收征管机制不断完善，财政与编制政务公开深入推进，行政服务中心效能进一步提高。土地收购储备和“招拍挂”出让制度全面建立，国有资产管理和城投公司的融资平台作用有效发挥。大力推进全民创业，当年新增个体私营企业1.8万户，新增注册资金53.9亿元。招商引资质效进一步提高。当年引进500万元以上项目538个，亿元以上项目57个，新开工外来投资项目526个，到位资金73亿元。外资外贸指标在全省双双进位，实际利用外资1.18亿美元，比上年增长25.5%;外贸出口3.62亿美元，比上年增长74.9%。对外交流与合作加强，与四川眉山、陕西汉中、湖南株洲等城市建立友城关系，与芬兰科沃拉市建立友好合作关系。各级开发区(工业园区)在改革开放、招商引资等方面迈出较大步伐。

　　人民生活有了新改善。实施积极的就业政策，当年新增城镇就业4.7万人。社会保险扩面16.4万人，征收社会保险费10.5亿元。连续3年调高企业离退休人员的养老金待遇，养老金人均月增加218元，增长40%。4.6万名国有困难企业退休人员和15.6万名城镇低保对象全部纳入了基本医疗保险。农村低保工作全面启动，18万名农村特困救助对象首批受益。加大扶贫力度，农村绝对贫困人口减少5万人，低收入贫困人口减少8万人。多途径组织残疾人就业，妥善安排农村“五保”老人生活。切实维护群众的合法权益，加强农民负担监管，综合治理拖欠农民工工资和医疗教育领域的乱收费问题。全市城镇居民人均可支配收入8314元，比上年增长19.1%;农民人均纯收入3295元，比上年增长15.2%。

　　社会事业取得新进步。全市财政用于科技、教育、文化、卫生方面的支出23.2亿元，比上年增长41.8%。着力打造黄冈教育品牌，建立农村义务教育经费保障机制，落实农村义务教育阶段学生“两免一补”资金近3亿元，基础教育、职业教育、高等教育均衡发展。成功举办了全国基础教育·黄冈论坛、湖北省第六届黄梅戏艺术节和黄冈市第二届运动会。科技创新能力增强，“市校合作”稳步推进，科技特派员工作全面开展。我市首次荣获“全国科技进步先进市”称号。加强公共卫生体系建设，新型农村合作医疗参合农民483.2万人，参合率86.6%。艾滋病、血吸虫病等重大传染病和地方病防治取得新进展。深入开展“关爱女孩行动”，综合治理出生人口性别比偏高问题。加大了土地、水、森林、矿产等资源保护力度。加强节能减排和环境保护，治理了15户污染企业，关闭拆除了3家小水泥和11家小造纸企业。全市化学需氧量和二氧化硫排放量分别削减2.8%和2.65%，亿元GDP综合能耗下降2.6%。认真落实安全生产责任制，加强隐患排查整治，安全生产各项指标都控制在省政府下达的责任目标范围内。社会消防工作连续五年受到省政府表彰。产品质量和食品药品安全监管等工作取得明显成效。

　　民主法制和精神文明建设得到新加强。深入贯彻依法治市方略，加强法制宣传教育，推进基层民主建设，村务公开和村民自治制度不断健全，城市社区居民自治和基层管理体制得到完善，行政复议和行政应诉综合试点工作取得阶段性成果。加强社会治安综合治理和“平安黄冈”建设，依法严厉打击各类犯罪，保持了社会稳定。加强社会主义核心价值体系和思想道德建设，深入开展群众性精神文明创建活动，公民思想道德素质和城乡文明程度得到提高。加强革命传统教育基地建设，隆重举行了黄麻起义80周年纪念活动。积极开展国防动员和双拥工作，我市首次被命名为“全国双拥模范城”。

　　民族、宗教、外事、侨务、对台、人防、信访、保密、档案、气象、地震、方志、经研、妇女、儿童、老龄等各项工作都取得了新的成绩。

　　过去的一年，我们围绕科学民主决策、推行依法行政和狠抓工作落实，全面履行政府职能，着力加强思想建设、队伍建设、作风建设、制度建设和廉政建设，加强社会管理和公共服务，建立应对突发公共事件的管理体制机制，加快电子政务建设，加强监察和审计工作，加大反腐倡廉力度，政府自身改革和建设迈出了新的步伐。

　　在市三届人大一次会议上，市政府承诺办好8个方面的实事。经过一年来的努力，除市级行政中心东移工程因国家政策所限尚未实施外，其他均已圆满完成或超额完成。其中，通村公路、饮水安全、乡村宽带、新型农村合作医疗、农村寄宿制中小学改造和劳动力就业培训转移等项目超额完成任务，农村低保、水库移民、廉租住房等政策全面落实，市区惠民医院投入运营、社区卫生服务试点扎实推进，市直国有困难企业职工养老保险和退休人员医疗保险问题妥善解决，市区城市建设目标如期实现，黄冈市儿童福利中心动工建设。

　　各位代表!过去一年我市经济社会发展取得的成绩，是市委正确领导，全市人民同心同德、艰苦努力的结果;是市人大、市政协监督支持，各民主党派、工商联、无党派人士，各人民团体，中国人民解放军驻黄部队、武警部队，海内外黄冈同乡、各界朋友和投资客商大力支持的结果。在此，我代表市人民政府，向全市广大干部群众、向所有关心支持黄冈发展的海内外人士，表示衷心的感谢!

　　总结过去一年的工作，我们清醒地认识到，政府工作与上级的要求和全市人民的期待还有较大差距，经济社会发展中仍存在不少矛盾和问题。主要是：经济发展速度不快与质效不高的问题并存，经济总量偏小、人均水平较低、产业结构不优，长期以来形成的结构性矛盾和粗放型增长方式尚未根本改变;农民持续稳定增收的渠道不宽，农村生产生活条件亟需进一步改善;社会事业改革和发展相对滞后，社会保障体系还不够健全，一些关系群众切身利益的问题需要继续下大力解决;政府自身改革和建设有待进一步加强，一些部门办事效率不高，有些政府工作人员服务意识和依法行政观念不强、作风不实，甚至为政不廉，等等。对此，我们必须以实事求是的态度，采取切实可行的措施，认真加以解决。

　　二、2008年政府工作的指导思想和奋斗目标

　　2008年是全面贯彻落实党的十七大精神的第一年，是实施“十一五”规划承上启下的一年，是武汉城市圈资源节约型和环境友好型社会建设综合配套改革试验区的开局之年。做好今年的工作，具有十分重要的意义。今年政府工作的指导思想是：全面贯彻落实党的十七大精神，以邓小平理论和“三个代表”重要思想为指导，以科学发展观统领经济社会发展全局，继续坚持“四抓”的工作思路，进一步解放思想和转变经济发展方式，积极推进改革开放和自主创新，突出抓好招商引资和全民创业，切实加强节能减排和环境保护，更加重视改善民生和促进社会和谐，统筹经济建设、政治建设、文化建设、社会建设，推动经济社会又好又快发展。

　　今年经济和社会发展的预期目标是：生产总值增长11%以上，全社会固定资产投资增长20%以上，社会消费品零售总额增长15%左右，吸收外商直接投资增长12%左右，外贸出口增长15%以上，财政收入增长20%以上，其中地方一般预算收入增长16%以上;单位生产总值能耗下降4.4%，化学需氧量和二氧化硫排放总量分别减少1%;城镇登记失业率控制在4.5%以内，城镇居民人均可支配收入增长10%以上，农民人均纯收入增长8%以上，居民消费价格涨幅控制在4.8%以内;人口出生率控制在11‰以内，亿元GDP生产安全事故死亡人数下降到0.5人以下。

　　上述经济社会发展目标，是综合考虑各方面因素，根据需要和可能提出来的。实现这个目标，既有机遇又有挑战，机遇大于挑战。中央加大对中西部地区、革命老区、贫困地区和新农村建设的支持力度，有利于黄冈争取更多的政策、项目和资金;境外和沿海产业加速向中西部地区转移，黄冈的生态优势、后发优势和低成本优势明显，可望在新一轮竞争中抢占先机，争取主动，加快发展;武汉城市圈“两型社会”建设综合配套改革试验区的设立，为我们改革体制机制、转变发展方式、提高发展质量和效益提供了重大战略机遇;沪汉蓉快速铁路、大广高速、武英高速、武麻高速、鄂东大桥、黄州火车站升级改造等国家和省重点工程相继竣工或开工，使我市区位交通优势更加明显;经过多年的不懈努力，我市经济结构不断优化，发展后劲不断增强，招商引资和重点项目建设蓄积的能量开始释放。通过营造竞争环境、建立激励机制，市县之间、单位之间比学赶超、竞相发展，广大干部群众加快发展的积极性和创造性进一步增强。与此同时，中央实施稳健的财政政策和从紧的货币政策，严格控制货币信贷总量和投放节奏，严格控制新开工项目，客观上会给我们欠发达地区带来较大压力;资源环境约束加剧，区域竞争更趋激烈，能源原材料需求扩大、物价上行压力增加，以及刚刚遭遇历史罕见的冰雪灾害，等等，可能给经济社会发展带来不确定和不稳定因素。全市上下务必强化忧患意识、责任意识和竞争意识，坚持科学发展、和谐发展、抢先发展，充分调动一切积极因素，千方百计保持较快的增长速度，以快求好、以好保快，努力开创黄冈改革发展的新局面。

　　三、着力转变经济发展方式，促进经济又好又快发展

　　(一)加快推进新型工业化

　　坚持重点抓工业，实施100亿元产业、100亿元工业园区、100家重点企业、100个重点项目、新增100家规模以上企业、市县领导联系100家民营企业等“六个一百工程”，力争规模以上工业增加值突破100亿元，增长20%以上。

　　着力培育产业集群。重点抓好食品饮料、纺织服装、医药化工、机械电子、建筑建材等产业集群，在优化结构、提高质量，降低能源消耗和减少污染物排放的基础上，扩大产能，延伸产业链，提高产业支撑和带动力。集中力量抓好一批骨干企业，支持100家重点企业推进资源整合、增资扩股，力争年产值过亿元企业达到50家以上、过10亿元企业达到10家以上。继续实施“中小企业成长工程”，建立中小企业服务体系，加大扶持力度，促进中小企业不断提高技术和管理水平，增强发展活力与市场竞争力。实施精品名牌战略，争取再有一批产品跻身中国名牌、中国驰名商标和中国出口名牌，培育发展一批地方名牌。

　　加快开发区和工业园区建设。进一步理顺和创新开发区(工业园区)体制机制，加强园区招商，推进工业项目向园区集中，以项目落户推动园区建设，以园区建设促进招商引资。突出园区的产业特色，提高园区产业配套能力，力争产值过100亿元的工业园区建设有较大突破。

　　强力推动企业上市。加强与专业机构的联动与合作，积极推动本地优势企业和成长性好的企业上市。对列入上市预备梯队的企业在税费、融资等方面加大扶持力度，使之尽快达到上市条件。争取当年上市企业达到2家以上。

　　加快企业信息化。引导和鼓励企业采用先进适用的信息技术改造提升传统产业，推动生产制造自动化、产品智能化、经营管理网络化以及商务电子化，使企业在集成化、数字化、网络化的平台上实现产品的优化设计、快速制造，不断提升企业信息化水平，增强企业的生存能力和市场竞争能力。

　　加强企业家队伍建设。制定和落实对企业家的激励政策，加强教育和管理，加大培养与引进力度，在全社会营造“鼓励竞争，激励创新，崇尚成功，宽容失败”的良好氛围。

　　(二)进一步加强社会主义新农村建设

　　大力发展现代农业。坚持以工业理念谋划农业，以农产品加工业带动基地建设，进一步加大龙头企业培育力度，强力推进农业领域尤其是畜禽加工行业的招商引资工作，着力引进和培育一批产值过亿元的大龙头。依托伊利、太子奶集团，加快奶牛养殖小区和牧场建设，确保每个县市区建好两个以上千头奶牛牧场，存栏奶牛总量达到2万头;依托林纸加工企业，推进速生丰产林建设，加强低产林改造，扩大林业基地规模，确保林纸一体化项目基地达到450万亩;依托神鹭、海鲲集团，加强小龙虾苗种繁育基地建设，力争小龙虾养殖面积发展到30万亩。继续推进优势农产品板块基地建设，认真落实支农惠农政策，在稳定粮食、棉花生产的同时，加快建设畜牧、水产大县和双低油菜基地。加强农业科技推广工作，抓好“科技入户工程”。加强动物疫病和植物病虫害防控，努力减少灾害损失。加大农业标准化和机械化扶持力度，提高农产品质量，促进农业增效增收。

　　加强农村基础设施建设。保质保量按时完成长江回水堤防建设工程，更新改造9座大型泵站，除险加固15座小型病险水库，完成蕲水灌区续建工程。进一步抓好高产农田建设，继续实施退耕还林，治理水土流失面积100平方公里。加快农村饮水安全、电网、通信、沼气等工程建设，解决44.13万农村人口饮水安全问题，完成农村沼气“一建三改”项目5.25万户。加强农村商业、文化等设施建设，新建和改造农家店600家，完成20户以上自然村广播电视村村通工程。新农村建设试点范围扩大到20个乡镇、200个村。

　　加大扶贫开发和灾后重建力度。继续实施“1321”工程，实现137个重点贫困村和62个老区贫困村的整村推进目标。整合扶贫资源，确保当年解决7万绝对贫困人口和12万低收入人口的温饱与脱贫问题。切实加强灾后重建和恢复生产工作，千方百计把冰雪灾害造成的损失降低到最低限度。

　　(三)大力发展现代服务业

　　加快发展金融保险业。加强征信体系建设，培植优质信用主体，完善信用担保和中介服务体系，进一步加强金融生态环境建设。健全信贷激励机制，推动金融业务创新，加大信贷投入，提高金融业经营水平和效益。支持商业保险机构积极拓展“三农”保险，发展各类责任保险、财产保险和人身保险。精心培育旅游品牌。把旅游业作为优先发展的重点产业之一，支持各类市场主体参与旅游开发，加快精品旅游线路、重点景区和旅游公路、服务设施建设。支持“农家乐”等旅游项目发展。加强旅游宣传营销工作，精心组织节会活动，提高黄冈旅游知名度。积极发展房地产业。认真实施住房建设规划和房地产用地统一储备供应制度，支持规模开发和集中连片住宅小区建设。调整住房供应结构，健全住房公积金制度，加快经济适用住房和廉租住房建设，防止房价过快上涨，逐步解决低收入家庭住房困难。严格控制零星分散建设，加大违法建设查处力度。规范物业管理服务，提升居住环境质量。着力培育黄冈教育品牌。加强黄冈教育品牌的培育、推介、管理和保护力度，增创黄冈教育发展新优势。推动教育资源尤其是职业教育资源有效整合，扩大教育规模，提升办学质量。改造提升传统服务业。以连锁经营、统一配送、现代物流和电子商务等现代流通形式，改造提升商贸、餐饮、交通运输等传统服务业，提高传统服务业的组织化、现代化水平和市场竞争力。加大龙头商贸企业培植力度，抓好重点专业批发市场的培育，推进“万村千乡市场工程”建设。

　　(四)加快重点项目建设

　　把保持投资增速与优化投资结构有机结合起来，千方百计争取国家项目和资金，千方百计招商引资，千方百计加快项目建设。力争当年固定资产投资突破310亿元。交通方面，加快沪汉蓉快速铁路黄冈段、大广高速黄冈段、武英高速黄冈段、武麻高速红安连接线建设步伐，开工建设武麻高速公路，力争完成黄上公路改造工程。启动黄州火车站改扩建项目。全力支持京九铁路黄冈段电气化改造工程、中石化“川气东送”和中石油“西气东输”黄冈段管道建设工程。能源方面，加快大别山电厂、白莲河抽水蓄能电站建设，新上英山地热水能开发项目。积极做好大别山电厂二期、黄冈火电厂和龙感湖风力发电、浠水太阳能级多晶硅生产线等项目的前期工作。工业方面，实施贝因美(黄冈)食品加工、亚东(黄冈)水泥、华新(麻城)水泥、鸿路(团风)钢构、稻花香(黄梅)酒业、武穴祥云二期、罗田新世纪纺织二期、蕲春李时珍药业二期等148个重点项目。加快推进林纸一体化项目建设。市政设施建设方面，加快城市道路建设和改造步伐，建设完善城市排水、供水、供气设施，提高城镇的综合功能。

　　(五)统筹城乡协调发展

　　加快市区发展，确保主要经济指标增幅高于全市平均水平。着力抓好工业园区和重点工业项目建设，确保新增工业产值30亿元以上。深入开展“四城联创”，实施遗爱湖治理保护、主干道路黑化硬化、园林绿化、市政设施配套等重点工程。加快实施旧城改造。抓好城区“菜篮子”工程，力争扩大基地面积3万亩。认真贯彻“一主三化”方针，发挥比较优势，壮大主导产业，增创特色优势，推进县域经济上规模、上水平、上台阶。完善激励竞争机制，鼓励支持基础较好的县市率先建成经济强县，提升在全省县域经济中的位次。加强城乡规划、建设和管理，树立经营城市理念，完善县市城区功能，扩大中心城镇规模，增强城镇的辐射带动作用。

　　(六)强化资源节约和生态环境保护

　　进一步强化生态文明理念，积极创建生态县市、生态村镇、生态园区和清洁家园、清洁田园、清洁水源。深入开展整治违法排污企业专项行动，突出抓好医药、化工、纺织、建筑、建材等重点行业、重点领域节能减排工作，坚决关闭工艺落后、设备老化、污染严重的企业。对政府、企业和相关单位实行严格的节能减排工作责任制、问责制和“一票否决”制。严把项目准入关。把投资强度、产出效益和生态环保、资源利用，作为选择项目、招商引资的重要指标，新上项目坚决进行环评和落实“三同时”，决不引进破坏资源、恶化环境的项目。加快城市污水处理厂和垃圾处理场建设与运营管理，黄冈市区和武穴、麻城污水处理厂确保今年建成投入使用，污水处理率达到60%以上;其他县城污水处理厂年内开工建设。加强耕地保护和土地集约节约利用。落实耕地保护行政首长负责制，按照开发整理造地、集约节约用地、重点保障工业项目用地的原则，严格执行土地利用总体规划和年度计划。加强荒地开发、废弃地整理和迁村腾地，继续开展市区和农村个人建房用地清查处理工作。

　　四、推进改革开放与自主创新，完善推动科学发展的体制机制

　　积极投入武汉城市圈“两型社会”综合配套改革试验区建设。按照加快产业布局、基础设施、市场体系、城乡建设、生态环境五个一体化的思路，着眼于体制机制创新，在重点领域和关键环节大胆实验，率先突破，切实走出一条新型工业化、新型城市化发展新路。充分发挥黄冈的比较优势，积极承接圈内产业转移，不断深化工业、农业、旅游、劳务等方面的协作配套，形成在城市圈内分工合理、特色鲜明、优势互补、聚集发展的产业格局，在区域经济融合中加快发展、奋力赶超。

　　继续加大招商引资力度。进一步拓宽思路，实行全方位、多领域招商，在突出重点引进工业项目的同时，注重引进现代农业项目、服务业项目和社会事业项目，实现经济社会协调发展。创新招商方式，积极适应沿海产业以产业链条为纽带的整体转移和配套转移的新特点，大力推进产业集群招商，实现由“单个企业招商”向“产业整体引进”转变，从“承接产业”到“对接产业”转变。精心组织重大招商引资活动，积极参与第三届中国中部投资贸易博览会。

　　加强对外经贸工作。坚持引商引资引智和外资外经外贸并重，在扩大利用外资规模，提高外资利用质量的同时，进一步培育出口品牌，扩大出口规模，提高出口效益。重点抓好机电产品、高新技术产品、纺织服装、医药化工和农产品出口。加强外经外贸和外汇服务创新，积极推进黄冈贸易投资便利化。加快实施“走出去”战略，加强对外工程承包、服务外包、海员外派和经济技术协作。

　　进一步深化各项改革。继续推进商贸企业和部门企业改革。巩固农村综合改革成果，进一步完善农村公益性服务“以钱养事”新机制。全面推进集体林权制度改革。大力发展农民专业合作组织，提高农民组织化程度。积极探索土地有序流转，推进土地适度规模经营。加快建立国有资本经营预算制度。加强机构编制管理，完成县属事业单位改革任务，确保乡镇机构编制只减不增。深化财政管理体制改革，完善部门预算、国库集中支付、政府采购、财政与编制公开改革，加强非税收入管理，推广公务卡结算方式。坚持依法治税，强化税收征管，落实各项税收政策，确保均衡入库、应收尽收。

　　大力推进全民创业。完善和落实促进全民创业的政策措施，形成有利于各类市场主体创业的体制机制，支持城乡居民、复员退伍军人和大中专毕业生等自主创业。毫不动摇地鼓励引导非公有制经济发展，支持非公有制经济进入公用事业、基础设施以及社会事业等领域，扶持重点民营企业做大做强。

　　着力提高自主创新能力。培育创业创新文化，强化企业主体地位，完善鼓励技术创新和科技成果产业化的激励机制与市场环境，深化市校、企校合作，积极争取国家和省科技项目，促进科技与经济发展的紧密结合。实施科技创新引导工程，引导和支持创新要素向企业集聚，促进创新成果直接向现实生产力转化。

　　五、加快发展各项社会事业，推进和谐黄冈建设

　　大力发展教育、文化、卫生等社会事业。进一步完善义务教育经费保障机制，为农村义务教育阶段学生免费提供教科书，提高农村家庭经济困难寄宿生生活费补助标准和农村中小学公用经费标准;从今年秋季学期开始，全部免除城市义务教育阶段学生学杂费。进一步提高对中等职业学校贫困家庭学生免除学杂费的比例。支持高校提高办学能力。全面推进新型农村合作医疗制度建设，实现以县市区为单位全覆盖。稳步实施医药卫生体制改革，加强惠民医院、社区卫生服务站和乡镇卫生院、村级卫生室建设，抓好艾滋病、血吸虫病、结核病、人感染高致病性禽流感等重大传染病防治。加强公共文化设施建设，实施乡镇综合文化站建设、农村电影放映和农家书屋工程。扶植文化市场主体，支持黄梅戏、出版、报业、娱乐等产业加快发展。转变广播电视运营方式，加快有线电视网络由模拟向数字化整体转换。加强二轮修志工作。突出“迎奥运”主题，广泛开展群众性体育活动，培养后备人才，提高竞技水平。建立健全关爱女孩及计划生育利益导向机制，稳定低生育水平，提高出生人口素质，确保符合政策生育率达到90%以上，出生人口性别比稳步下降。认真做好第一次污染源普查、第二次经济普查、第二次土地调查和第三次文物普查工作。

　　努力做好就业和社会保障工作。进一步完善市场就业机制，以发展增加就业，以创业带动就业，确保城镇新增就业4.1万人。健全创业服务体系和职业培训制度，完善和落实就业再就业扶持政策，扎实开展面向所有困难群众的就业援助工作，及时帮助零就业家庭和失地农民解决就业问题。加快建立覆盖城乡居民的社会保障体系，全市城镇职工基本养老保险达到44万人，城镇职工基本医疗保险达到39万人。加快城乡特殊困难群众社会救助体系建设，完善城镇和农村低保、农村“五保户”供养、特困户救助、灾民救济等制度。

　　高度重视社会管理和公共服务。认真落实信访工作领导包案制和工作责任制，积极化解各类矛盾。加强国家安全工作，防范和打击各种敌对势力的渗透破坏活动。加强社会治安综合治理，依法严厉打击各种违法犯罪，着力整治治安突出问题，保障人民群众安居乐业。加强安全生产基础工作和安全生产法制建设，层层落实安全生产责任制，努力减少较大事故，杜绝重特大事故。重视和加强社会消防工作。加大公路治超力度，确保道路交通安全畅通。深入开展食品安全专项整治行动，强化食品、药品、餐饮卫生监管，保障人民群众的健康安全。加强气象公共服务，扩大气象灾害预警信息覆盖面。加强价格监测、预警和监管，抓好“米袋子”、“菜篮子”建设，依法打击串通涨价、哄抬物价等各种价格违法违规行为，努力保持居民消费价格水平基本稳定。落实对低收入群众的补助办法，确保他们的基本生活水平不因物价上涨而下降。

　　积极支持部队和国防建设。关心和支持驻黄解放军、武警部队建设，深入开展国防教育，健全国防动员体系，抓好民兵预备役和人民防空工作。深入开展双拥共建活动，巩固创建“全国双拥模范城”成果。积极做好军转干部、复员退伍军人优抚和安置政策落实工作。

　　认真做好民族、宗教、外事、侨务、对台等工作。全面贯彻党和国家的民族政策，依法管理宗教事务。充分发挥外事、侨务等部门的职能作用，扩大对外交往与合作。进一步做好对台工作。努力解决农民工子女、留守儿童和社会弱势群体生活困难，关心青少年健康成长。积极发展残疾人事业，提高残疾人康复、教育、就业水平。关心和支持工会、妇女、老龄等事业发展。认真做好保密、档案等工作。

　　坚持不懈为群众办实事。在全面推进各项工作的过程中，把为民办实事放在更加突出的位置，加大财政投入和政策倾斜力度，努力办好就业、就学、就医、出行、住房等八个方面的实事。　　一是帮助1万人实现创业并带动3万人就业再就业;完成劳动力职业培训30万人次、转移培训农村劳动力11.5万人。二是建立被征地农民培训就业和社会保障制度;实施灵活就业人员养老保险补贴办法和基本医疗保险优惠政策。三是加快农村公路建设步伐，完善农村客运市场体系。当年建设农村公路2000公里、客运五级站7个、候车亭280个、招呼站560个，新增农村客运班线27条。四是从今年起用两年时间，对全市715所农村困难学校进行全面改造。当年建成农村合格学校200所。五是支持法律救助机构为经济困难群众和农民工无偿办理法律援助案件1000件以上，其他法律援助事项1万件以上。六是健全市区并启动麻城、武穴城市社区卫生服务体系建设，城市社区卫生服务覆盖率达到70%以上。七是全市建设廉租住房1.68万平方米、经济适用房25万平方米。八是加快推进遗爱湖治理保护工程，完成市区道路改造刷黑工程22万平方米，新建公厕20座。

　　六、进一步加强政府自身建设

　　围绕转变职能、理顺关系、优化结构、提高效能，更好地实践为人民服务的根本宗旨，不断提高政府管理经济和社会事务的能力，不断提高人民群众对政府的满意度和信任度，努力做到一切属于人民，一切为了人民，一切依靠人民，一切归功于人民。

　　切实提高政府执行力。坚持市委统一领导，认真执行人大及其常委会的各项决议，依法自觉接受监督;主动听取人民政协的意见与建议，积极支持政协履行职能。切实做好人大代表建议和政协委员提案办理工作。强化政府及其部门的工作责任，健全目标管理制度、绩效评估制度和竞争激励机制，严格落实行政首长负责制和行政问责制，全面推行岗位责任制、服务承诺制和限时办结制，加大行政监察和工作督查力度，形成崇尚实干、狠抓落实的发展氛围。进一步强化机关效能建设，坚持高标准、严要求，保持高效率、快节奏，工作程序简化、召开会议简短、文字材料简练、汇报工作简洁、语言表达简明、接待应酬简省、生活待遇简朴、深入基层简从，把更多的时间和精力用于深入基层调查研究、解决问题、推动落实。

　　切实提高政府创新力。创新行政理念，正确运用行政权力，转变观念，统筹资源，优化流程，提高效能，确保政府一切工作服从服务于发展的需要、服从服务于公平与效率的需要、服从服务于改善民生的需要。尤其对各类市场主体要精心培育、倍加呵护，搭建充分发展的平台。创新行政方式，积极探索建立科学的考核评价体系，完善工作量化考核办法。加强电子政务建设，完善政府信息公布制度，提高政府工作透明度和办事效率。加强应急管理工作，建立完善覆盖乡镇、社区和企事业单位的突发公共事件应急预案体系，推进应急管理体制、机制、法制建设，提高政府应对突发公共事件的能力。优化公共资源配置，加强公共服务平台建设，完善社会管理制度，提高社会管理水平。创新工作方法，坚持不懈抓重点、攻难点，善于总结经验，以点带面，推动全局。凡是有利于经济社会发展、有利于人民生活水平提高、有利于维护地区稳定的事，一定要敢抓敢管、着力推进、重点突破。

　　切实提高政府公信力。坚持依法行政，深入开展普法教育，严格行政执法责任制度和执法过错责任追究制度，加强评议考核，规范执法行为，促进严格、公正、文明执法。坚持阳光行政，进一步完善公众参与、专家论证和政府决策相结合的决策机制，建立政府新闻发言人制度和涉及群众切身利益的重大决策群众参与制度，健全市政府领导定期听取人大代表、政协委员意见制度，认真听取各民主党派、工商联和各人民团体的意见，自觉接受新闻舆论和群众监督。政府出台的政策、作出的承诺必须兑现，做到言必信、行必果。坚持廉洁从政，深入开展廉政建设和反腐败斗争，加大行政监察、审计监督和行风建设力度，全面落实建设工程招投标制度、经营性土地使用权“招拍挂”出让制度、产权交易制度和政府采购制度，规范市场交易行为，严肃查处违法违纪案件，努力建设一支廉洁奉公、勤政务实、作风优良的公务员队伍。

　　各位代表!新的一年，我们肩负的任务光荣而艰巨，我们面临的机遇宝贵而难得。让我们紧密团结在以胡锦涛同志为总书记的党中央周围，在市委的坚强领导下，深入贯彻落实科学发展观，积极进取，扎实工作，为实现经济社会又好又快发展、谱写人民美好生活新篇章而努力奋斗!
